# Letter to the Editor on: “Effect of Topical Application of Antimicrobial Peptide PL‐5 (Peceleganan) Spray on Mild to Moderate Infection of Diabetic Foot Ulcers: A Multicenter, Randomized, Double‐ Blinded, Placebo‐Controlled Clinical Trial”

**DOI:** 10.1111/1753-0407.70270

**Published:** 2026-09-15

**Authors:** Xiang Ma, Qingqing Shan

**Affiliations:** ^1^ Department of Respiration Chengdu Integrated TCM & Western Medicine Hospital Chengdu China

To the Editor,

We read with great interest the multicenter, randomized, double‐blind, placebo‐controlled trial by Qian et al. evaluating the topical antimicrobial peptide PL‐5 spray for mild‐to‐moderate diabetic foot ulcer infections [1]. The study's finding of superior clinical response rates and promising clearance of drug‐resistant bacteria represents a potentially valuable contribution to wound care. However, we wish to raise several methodological concerns that may affect the interpretation of the results.

First, we are concerned about the baseline imbalance in wound infection scores between groups. As shown in Table 2, the placebo group had a significantly higher median infection score at baseline (3.0 vs. 2.5, *p* = 0.0073), showing more severe infection at enrollment. While the authors attempted to adjust for this using a mixed‐effects model, statistical adjustment cannot fully eliminate the inherent bias introduced by baseline prognostic imbalance in a randomized trial [2]. This variation suggests that randomization may not have been stratified by infection severity, which may be compromising the comparability of the two groups. Given that prognosis is strongly correlated with baseline infection severity, this design flaw fundamentally compromises the robustness of the primary endpoint conclusion [3]. As Coskinas et al. demonstrated, post hoc decisions to adjust for observed imbalances risk inflating the probability of falsely declaring treatment superiority [4].

Second, the primary efficacy endpoint—defined as a ≥ 50% reduction in wound infection score—provokes concerns about subjectivity. The composite score includes components such as erythema, induration, tenderness, and pain, which are intrinsically subjective and susceptible to assessment bias [5]. Although the trial was double‐blinded, blinding is less effective in controlling bias for subjective outcomes than for objective ones [6]. The absence of objective inflammatory markers (e.g., CRP, PCT) as corroborative evidence further weakens the validity of the primary endpoint [7].

Third, the use of a placebo control in this context raises both ethical and clinical questions. Given that standard topical antimicrobials (e.g., silver sulfadiazine, mupirocin) are widely available, placebo‐controlled designs may expose patients in the control group to inferior care [8]. Furthermore, clinicians are more interested in knowing whether PL‐5 offers advantages over existing treatments rather than over placebo. The absence of an active comparator limits the clinical significance of the findings.

Fourth, the details of the screening failure warrant clarification. Figure 1 indicates that 12 of 60 screened patients did not enroll, of whom 10 did not meet the inclusion criteria. However, the specific reasons for these exclusions—whether due to infection severity, glycemic control, vascular disease, or other factors—were not reported. This information is critical for assessing the external validity of the trial and understanding to which patient populations the results can be generalized [9, 10].

We acknowledge the authors' contribution to this important field and the transparency with which baseline data were presented. Nevertheless, these methodological considerations call for careful attention when interpreting the study's conclusions. We would appreciate the authors' answer to these concerns.

## Author Contributions

**Xiang Ma:** supervision, writing – review and editing. **Qingqing Shan:** conceptualization, writing – original draft.

## Funding

The authors have nothing to report.

## Ethics Statement

The authors have nothing to report.

## Consent

The authors have nothing to report.

## Conflicts of Interest

The authors declare no conflicts of interest.

## Data Availability

Data sharing is not applicable to this article as no datasets were generated or analysed during the current study.
